# Enhanced effect of microdystrophin gene transfection by HSV-VP22 mediated intercellular protein transport

**DOI:** 10.1186/1471-2202-8-50

**Published:** 2007-07-08

**Authors:** Fu Xiong, Shaobo Xiao, Meijuan Yu, Wanyi Li, Hui Zheng, Yanchang Shang, Funing Peng, Cuiping Zhao, Wenliang Zhou, Huanchun Chen, Liurong Fang, Jeffrey S Chamberlain, Cheng Zhang

**Affiliations:** 1 Department of Neurology, First Affiliated Hospital, Sun Yat-sen University, Guangzhou 510080, P.R.China; 2 Center for Stem Cell Biology and Tissue Engineering of Sun Yat-sen University, Guangzhou 510080, P.R.China; 3 State Key Laboratory of Agricultural Microbiology, Huazhong Agricultural University, Wuhan 430070, P.R.China; 4 Lab Of Physiology, Life sciences School of Sun Yat-sen University, Guangzhou 510080, P.R.China; 5 Department of Neurology, University of Washington School of Medicine, Seattle, WA 98195-7720, USA

## Abstract

**Background:**

Duchenne musclar dystrophy (DMD) is an X-linked recessive disease caused by mutations of dystrophin gene, there is no effective treatment for this disorder at present. Plasmid-mediated gene therapy is a promising therapeutical approach for the treatment of DMD. One of the major issues with plasmid-mediated gene therapy for DMD is poor transfection efficiency and distribution. The herpes simplex virus protein VP22 has the capacity to spread from a primary transduced cell to surrounding cells and improve the outcome of gene transfer. To improve the efficiency of plasmid-mediated gene therapy and investigate the utility of the intercellular trafficking properties of VP22-linked protein for the treatment for DMD, expression vectors for C-terminal versions of VP22-microdystrophin fusion protein was constructed and the VP22-mediated shuttle effect was evaluated both *in vitro *and *in vivo*.

**Results:**

Our results clearly demonstrate that the VP22-microdystrophin fusion protein could transport into C2C12 cells from 3T3 cells, moreover, the VP22-microdystrophin fusion protein enhanced greatly the amount of microdystrophin that accumulated following microdystrophin gene transfer in both transfected 3T3 cells and in the muscles of dystrophin-deficient (*mdx*) mice.

**Conclusion:**

These results highlight the efficiency of the VP22-mediated intercellular protein delivery for potential therapy of DMD and suggested that protein transduction may be a potential and versatile tool to enhance the effects of gene delivery for somatic gene therapy of DMD.

## Background

**D**uchenne muscular dystrophy (DMD) is a lethal x-linked genetic disease characterized by progressive skeletal muscle degeneration, occuring in one in 3500 male live births [1]. DMD results from mutation in the dystrophin gene, leading to the absence of a functional dystrophin protein and loss of the dystrophin-glycoprotein complex (DGC), which together render the sarcolemmal membrane susceptible to mechanical injury [2,3]. These deficiencies in turn lead to chonic muscle fiber necrosis and diffuse wasting, with muscle weakness appearing in childhood, followed by severe parallesis in adolescence, and death in the 20 s [1]. In spite of the progress made in orthopedic, respiratory, and cardiac management of patients, there remains no effective treatment for this disease.

Replacing and/or repairing the mutated dystrophin gene by full-length or truncated versions of dystrophin is an attractive approach that may offer a treatment for DMD. Gene transfer of dystrophin into skeletal muscle has been accomplished by intramuscular or intravenous/arterial injection of a therapeutic plasmid [4-8] or viral vector [9-12]. Due to the potential for immune responses, the limited carrying capacity of viral vectors and the difficulty in specifically targeting muscles bodywide, viral vector-mediated DMD gene therapy needs more improvements before demonstrating clinical utility [13]. The simplest form of gene therapy is the direct injection plasmid DNA into skeletal muscle. Direct injection of plasmid DNA into *mdx *mice though intramuscular or intravenous/arterial injection can result in expression of dystrophin in up to 10% of the muscle fibers, leading to a decrease in the percentage of myofibers displaying central nucleation [4-8]. In a phase I clinical trial, nine patients were injected intramuscularly with a plasmid-vector encoding a dystrophin cDNA, and dystrophin expression was detected in six patients without any adverse effects [14]. These results are encouraging for the further development of this method, however, the poor transfection efficiency for plasmid-mediated gene therapy is still a problem, especially with a systemic muscle disease such as DMD. Thus, enhanced expression and increased production and distribution of dystrophin are likely to be important developments for plasmid-mediated gene therapy for DMD.

Recently, a class of proteins known as cell-penetrating peptides were reported that had the capacity to deliver their cargo molecules across plasma membrane bilayers and translocate into neighboring cells, thereby increasing intracellular delivery of various cargoes with molecular masses several times greater than their own. These proteins may enhance the low transduction efficiency typically obtained with plasmid-mediated gene therapy, and they include the Tat (transactivator of transcription) protein of human immunodeficiency virus (HIV)[15,16], the antennapedia homeodomain proteins (AntPs) of Drosophila melanogaster and other species [17,18], and VP22 of herpes simplex virus (HSV) [19]. Many experiments have demonstrated that VP22 can enhance intercellular trafficking of proteins fused to its N- or C-terminus *in vitro *and *in vivo *[20-26]. Moreover, VP22-fusion proteins retained both VP22-mediated transport properties and the biophysiological functions of the linked passenger proteins, enhancing the therapeutic effects of transduced genes and proteins. This property has been observed with VP22-linked p27, p53, CD and E2 for delivery to tumors [20-23], VP22-GFP following adenovirus-mediated transgene delivery to CNS neurons [24], and VP22-mediated intercellular protein delivery was useful in enhancing the efficacy of myocardial gene therapy [25]. VP22-*lac*Z transfection resulted in higher amounts of protein expression compared with *lac*Z transfection. Preclinical evaluation using the TK-VP22 fusion revealed an enhanced cytotoxicity *in vitro *and *in vivo *using mixed populations of wild-type and expressing cells [27-29]. However, there have been no reports about the application of protein transduction to Duchenne muscular dystrophy therapy. Here, we studied the translocation properties of the VP22 protein fused to a microdystrophin cDNA for potential therapy of DMD. We demonstrated that the HSV-1-derived VP22 protein enhanced microdystrophin protein accumulation *in vitro *and *in vivo*. Therefore, VP22 may be a potent and versatile tool to enhance the effectiveness of somatic gene therapy for DMD.

## Results

### VP22 mediated intercellular trafficking of microdystrophin fusion proteins in vitro

**Figure 1:**
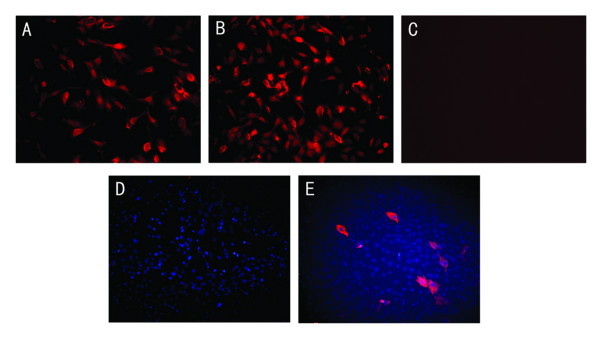
**Intercellular trafficking of VP22-MICDYS in the transfected C2C12 cells**. 3T3 cells were transfected with the plasmid pAMICDYS (A), pAVP22-MICDYS (B), pAVP22 (C) respectively. 48h post-transfection, transfected cells were fixed and immunocytochemistry was performed with the monoclonal anti-dystrophin Amino Terminal. Magnification: × 100. At 48 h after transfection with pAVP22, pAMICDYS, pAVP22-MICDYS, cell supernatant were prepared from 3T3 cells and then added to C2C12 cells in a 35 mm dish. After 45 min, the cells were washed with PBS and processed for immunocytochemistry. (D) Transfected by supernatant with pAMICDYS; (E) Transfected by supernatant with pAVP22-MICDYS. Magnification: × 200.

To determine whether the VP22-microdystrophin fusion proteins were able to spread from a primary expressing cell into neighboring cells, NIH 3T3 cells were transfected with 3.0 μg of the expression plasmids pAVP22, pAMICDYS, or pAVP22-MICDYS. 48 h post-transfection, indirect immunofluorescen -ce was performed. As shown in Fig. 1, in the wells transfected with pAMICDYS approximately 10% of cells were microdystrophin-positive after 48 h transfected; however, 47% of cells transfected with pAVP22-MICDYS were microdystrophin-positive, representing a 370% increase in the number of cells that contained microdystrophin protein. Following collection with the media supernatant and transfer onto the C2C12 myotubes to analyze whether HSV-VP22 is able to carry microdystrophin into C2C12 cells, indirect immunofluorescence was performed to detect the expression of microdystrophin after 45 min transfection (Fig. 1). As shown in Fig. 1D, some microdystrophin proteins were detected in C2C12 cells transfected by supernatant with pAVP22-MICDYS, the results clearly demonstrated that VP22 could transport the VP22-microdystrophin fusions from the primary infected cells (3T3 cells) into the C2C12 cells.

### VP22 enhanced microdystrophin gene expression and distribution in TA of mdx mice

**Figure 2:**
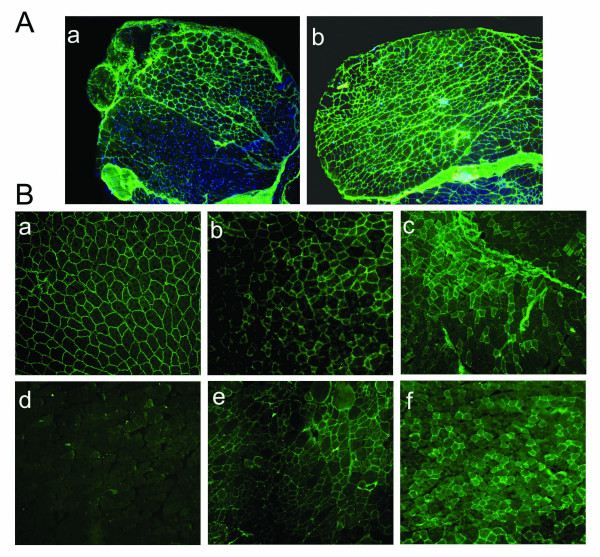
**VP22 enhanced microdystrophin gene expression and distribution in *mdx *mice**. (A) The effect of VP22 was reflected in the level of microdystrophin expression in pAMICDYS (a) and pAVP22-MICDYS (b) injected the left TA muscles. Microdystrophin -positive fibers were detected in both groups, but the number of microdystrophin-positive fibers and the intensity of immunofluorescence were uniformly greater in pAVP22-MICDYS injected TA muscles. Magnification: × 100. (B) Immunostaining analysis microdystrophin expression in *mdx *mouse TA injected by pAMICDYS (c, f) or pAVP22 -MICDYS (b, e) at 2 weeks (b, c) and 16 weeks (e, f) after injection, the right TA injected by PBS (d). The TA of C57BL/10 (a). Magnification: × 200.

**Figure 3:**
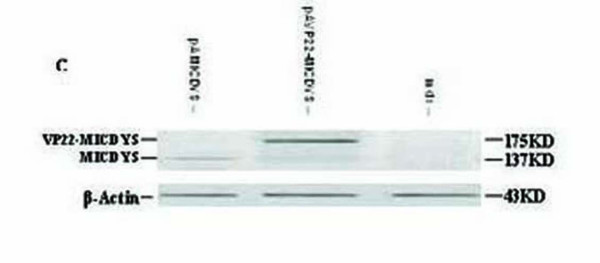
**Western blotting analysis indicated VP22 enhanced microdystrophin gene expression in TA of *mdx***. Western blotting analysis of microdystrophin expression in *mdx *TA injected by pAMICDYS or pAVP22-MICDYS after 2 weeks. The 175-kDa-microdystrophin bands appeared stronger in pAVP22-MICDYS- compared to pAMICDYS-transduced *mdx *TA.

To determine whether VP22 could enhance microdystrophin protein expression and whether the VP22-microdystrophin fusion proteins were able to spread in muscles of *mdx *mice, the plasmids pAMICDYS and pAVP22-MICDYS were injected intramuscularly into the TA muscles, and the expression of microdystrophin were tested by immunocytochemistry 2 weeks and 16 weeks post-injection and Western-blotting 2 weeks post-injection. As shown in Fig. 2A, microdystrophin-positive fibers per cross-section injected with pAVP22-MICDYS were markedly greater than that injected with pAMICDYS, moreover, there was no evidence of inflammation in the TA. However, there was a slight decline of microdystrophin-positive fibers in the TA from 2 weeks to 16 weeks after plasmids injection (Fig. 2B). Western-blotting analysis of microdystrophin gene expression was performed after 2 weeks following injection with equal dosage either pAMCIDYS or pAVP22-MICDYS (Fig. 3). Semiquantitative analysis of band intensity demonstrated that the expression of VP22-linked microdystrophin following pAVP22-MICDYS transfection was 3.1-fold greater than that of microdystrophin following pAMICDYS transfection. The number of microdystrophin-positive fibers per cross-section injected with pAVP22-MICDYS were significantly increased when compared to muscle of injected with pAMCIDYS. The average number of microdystrophin-positive fibers in each TA injected with pAVP22-MICDYS was about 2.3-times higher than that of TA injected with pAMCIDYS after 2 weeks (Table 1). Thus, VP22 fusion proteins increased the area containing dystrophin-positive muscle fibers to span almost all the left TA muscles and more than tripled the total number of TA myofibers transduced following a single microdystrophin injection.

**Table 1:** Quantitative analysis the numbers of microdystrophin-positive fibers of TA injected by pAMICDYS or pAVP22-MICDYS after 2 weeks

	N	Number of microdystrophin-postive fibers (SD ± mean)	Percent of total fibers
Control	8	2 ± 1	< 1%
pAMICDYS	10	64 ± 27	14 ± 3%
pAVP22-MICDYS	10	139 ± 32	32 ± 5%

N, Number of muscles used for the analysis in each group.

*, Significant different compared to pAMICDYS, *p *< 0.05.

### VP22-mediated microdystrophin gene expression is more efficient in correcting central nucleation in mdx muscle

**Figure 4:**
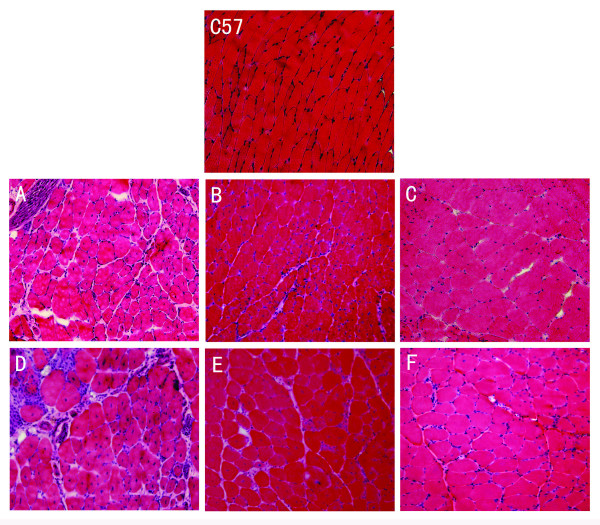
**VP22-mediated microdystrophin gene expression was more efficient in reducing central nucleation in *mdx *mice**. Histological analysis the left TA of mdx mice injected by pAMICDYS, pAVP22-MICDYS and the right TA injected by PBS. Shown are H&E-stained TA muscle cross sections from post-injection 2 weeks (A, B, C), post-injection 16 weeks (D, E, F). The left TA muscles treated by pAMICDYS (B, E), pAVP22-MICDYS (C, F); the right TA muscles treated by PBS (A, D). Magnification: × 200. Centrally nucleated fibers were counted in transverse H&E cryosections from treated mdx mice. The ratio of centrally nucleated fibers (%) is shown as means ± SE in TA muscle at post-injection 2 weeks (G) and 16 weeks (H). * p < 0.05 compared with *mdx *mice; ^+^p < 0.05 compared with pAMICDYS transfered mice.

**Figure 5:**
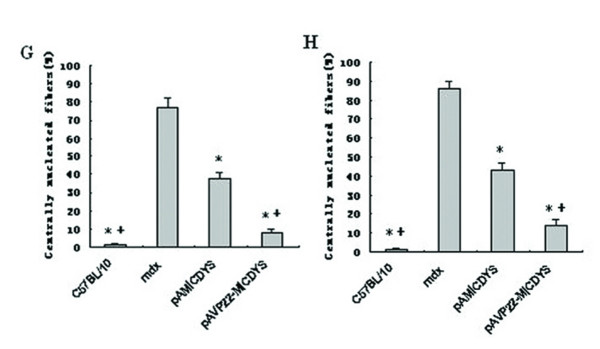
**The number of centrally nucleated fibers in treated TA of *mdx***. Centrally nucleated fibers were counted in transverse H&E cryosections from treated *mdx *mice. The ratio of centrally nucleated fibers (%) is shown as means ± SE in TA muscle at post-injection 2 weeks (G) and 16 weeks (H). * p < 0.05 compared with *mdx *mice; ^+ ^p < 0.05 compared with pAMICDYS transfered mice.

Muscles of *mdx *mice are characterized by the presence of abundant central nuclei in myofibers, and previous studies have demonstrated that transgenic expression of the microdystrophin in *mdx *mice remarkably ameliorated *mdx *mouse skeletal muscle pathology and the percentage of central nucleation was reduced to less than 1% in both limb muscle and the diaphragm, AAV-mediated microdystrophin expression also reduced central nucleation from 70% to 14% in 7-week-old mdx mice [30]. To further investigate whether fusion with VP22 can enhance microdystrophin spread, the degree of central nucleation in myofibers was monitored following injection of plasmid expressing VP22-microdystrophin or VP22 alone. HE staining evaluated the amelioration of muscle pathology (Fig. 4). Compared with contralateral muscles of *mdx *mice treated by PBS, the extent of central nucleation of *mdx *TA muscles injected with pAMICDYS was reduced from 77 ± 5% to 38 ± 3% (2 weeks post-injection) and 86 ± 4% to 43 ± 4% (16 weeks post-injection). However, compared with contralateral muscles of *mdx *mice treated by PBS, the number of centrally-located nuclei in pAVP22-MICDYS transduced TA muscles were significantly reduced from 77 ± 5% to 9 ± 2% (2 weeks post-injection) and 86 ± 4% to 14 ± 3% (16 weeks post-injection) (Fig. 5). These results indicated that spread of the microdystrophin fusion protein could retard significantly the degeneration of muscle in young mdx mice. Furthermore, VP22 enhanced the spread of microdystrophin and was more effective in reducing the number of centrally nucleated myofibers.

### VP22-microdystrophin fusion protein improved the distribution in the abdominal cavity and the contractile function of the diaphragm muscle with mdx mouse

**Figure 6:**
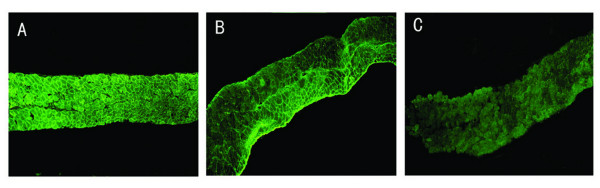
**Immunohistochemical staining of microdystrophin in the diaphragm muscle from *mdx *mouse injected intraperitoneally with pAMICDYS or pAVP22-MICDYS**. The diaphragm muscle with C57BL/10 mouse (A); the diaphragm muscle with *mdx *mouse treated by pAVP22-MICDYS (B); treated by pAMICDYS (C). Microdystrophin -positive fibers were only detected in groups injected by pAVP22-MICDYS, not in groups injected by pAMICDYS. Magnification: × 100.

**Figure 7:**
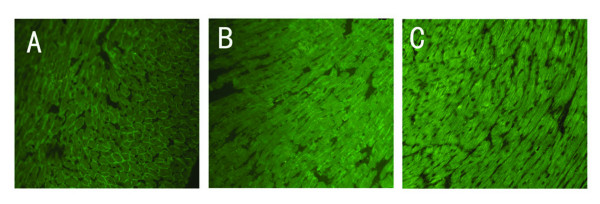
**Immunohistochemical staining of microdystrophin in myocardium from *mdx *mouse injected intraperitoneally with pAMICDYS or pAVP22-MICDYS**. The myocardium with C57BL/10 mouse (A); the myocardium with *mdx *mouse treated by pAVP22-MICDYS (B); treated by pAMICDYS (C). Microdystrophin -positive fibers were only detected in groups injected by pAVP22-MICDYS, not in groups injected by pAMICDYS. Magnification: × 100.

It has been reported that the protein transduction domain (PTDs) of the TAT-protein could mediate delivery of the biologically active fusion protein to all tissues in mice following intraperitoneal injection, portal vein injection, tail vein injection and after oral administration [31]. To observe whether VP22-mediated microdystrophin fusion protein also could spread in *vivo *and ameliorate the functional deficiencies of the diaphragm of *mdx *mice, the plasmids pAVP22, pAVP22-MICDYS or pAMICDYS were injected intraperitoneally in 6–8 week old *mdx *mice. Two weeks after injection, the expression of microdystrophin proteins was detected in myocardium, quadriceps and diaphragm muscles by immunohistochemistry. There were almost no microdystrophin-positive fibers in myocardium, quadriceps and diaphragm muscles after injection of pAVP22 or pAMICDYS, there were also no microdystrophin-positive fibers in quadriceps after injection of pAVP22-MICDYS. However, some microdystrophin-positive fibers were easily detected in diaphragm muscles (Fig. 6) and heart muscles (Fig. 7) after injection of pAVP22-MICDYS. To demonstrate if a functional recovery could be measured in the diaphragm muscle, the isometric contractile properties of diaphragm muscles were tested *in vitro*. Compared with muscles that were transfected with pAVP22, pAMICDYS or treated by PBS, VP22-microdystrophin expression led to better protection against eccentric contraction-induced injury (Table 2).

**Table 2:** Functional effects of pAMICDYS and pAVP22-MICDYS for diaphragm muscles though intraperitoneal injection

	C57BL/10	mdx	pAVP22	pAMICDYS	pAVP22-MICDYS
	
	(n = 4)	(n = 4)	(n = 3)	(n = 5)	(n = 5)
BM (g)	20.3 ± 1.5	23.8 ± 1.6	22.5 ± 1.5	23.3 ± 1.3	21.8 ± 1.6
sP_0 _(KN/m^2^)	215 ± 6.3	90.8 ± 4.3	92.1 ± 5.1	98.7 ± 5.8	145.3 ± 6.9*

Values are means ± SEM; n, number of mice; BM, body mass; sPo, specific maximum isometric tetanic force.

* P < 0.05 versus untreated *mdx *and treated by pAMICDYS.

## Discussion

Presently, gene therapy research for DMD focus mostly on viral or plasmid-mediated gene transfer methods. Although viral vectors have a higher transduction efficiency, they display several drawbacks, such as the limited carrying capacity of adeno-associated viral vectors, and the propensity of adenoviral, retroviral and herpes simplex viral vectors to elicit a strong immune response against either vector or transgene encoded proteins. Thus, all viral vectors are biological agents that carry at least some risk to patient safety. Compared to viral vectors, plasmid-mediated gene transfer has several advantages due to its simplicity, safety and reliability. Plasmid-mediated DNA transfer has no significant side effects and the highly purified, large-scale plasmid DNA can be easily obtained and the quality of plasmid DNA can be easily controlled [32]. Furthermore, DNA is much more stable than viral vectors and can be refrigerated for long periods of time. Exceptionally, it seems that skeletal muscles internalize plasmid DNA much more efficiently than other tissues do [33]. Therefore, plasmid DNA therapy may be more suitable for treatment of muscular dystrophy than viral vector mediated gene therapy. Unfortunately, the transfection efficiency of plasmid DNA by intramuscular injection is relatively low and transgene expression only occurs within a few centimeters from the injection site.

In this study, the HSV-1 VP22 protein was capable of mediating intercellular protein transport, significantly enhancing the transduction efficiency of plasmid expressing microdystrophin. To assess the feasiblity of application of VP22 in gene therapy of DMD, we first evaluated the VP22 shuttle effect in 3T3 cells and C2C12 cells *in vitro*. In imunofluorescence study, the VP22 fusion increased microdystrophin accumulation in 3T3 cells and transported microdystrophin into C2C12 cells. Following intramuscularly injection of the VP22-microdystrophin plasmid into the TA muscles of *mdx *mice, the VP22 fusion increased both expression and distribution of the VP22-microdystrophin fusion protein. Interestingly, microdystrophin mRNA was detected in some consecutive muscles adjacent to injected TA such as gastrocnemius and soleus muscles. One explanation is that the VP22 PTDs may carry the micelles of plasmid pAVP22-MICDYS into neighboring cells. Alternatively, PTDs may directly carry DNA into the neighboring cells. As a result, carried plasmid pAVP22-MICDYS transcript microdystrophin mRNA. However, naked plasmid DNA renders very low level of gene delivery by direct i.m, electroporation should be applied with i.m to enhance greatly the efficiency of VP22-mediated gene therapy.

Due to the absence of dystrophin, DMD patients develop progressive muscle degeneration in the diaphragm, which often leads to respiratory failure and death. Therefore, gene transfer into the diaphragm is important for the treatment of DMD patients. Microdystrophin has been shown to restore expression of the dystrophin-glycoprotein complex and improve sarcolemma integrity in the *mdx *mouse heart [34] and diaphragm [11,30]. However, delivery of the functional microdystrophin gene to diaphragm is a formidable problem. Although gene transfer into the diaphragm is readily obtained by either direct injection or pressure-mediated approach in experimental *mdx *mice [35], it is not feasible to apply those methods to patients. Protein transduction domains (PTD) can mediate efficient transduction of all tissues in mice, even the brain and skeletal muscle cells when administered by intraperitoneal or intravenous injection [31,35-38]. For example, intraperitoneal injection of a fusion protein encoding the 116-kDa β-galactosidase fused to the TAT PTD resulted in delivery of the biologically active fusion protein to all tissues in mice, including the diaphragm and the brain [31]. In the present study, expression of microdystrophin, along with a partial functional recovery of the diaphragm contractile properties was observed in diaphragm and heart muscle after intraperitioneal injection of fusion protein plasmid. We found fusion with VP22 could expand the distribution of the microdystrophin protein in the abdominal cavity and improved the contractile function with diaphragm. Moreover, the VP22-microdystrophin fusion protein could transport into heart muscle through diaphragm. We did not detect fusion protein in the brain or other skeletal muscles, suggesting that VP22 has only little capability to cross the barrier of blood vessel. This deficit of VP22 may also be improved by using a new type of PTD of VP22 with altered amino acid sequence, or by using a different PTD as the carrying vector. Alternatively, the purified fusion protein can be injected directly into the body though by intraperitoneal or intravenous injection.

In the present study, microdystrophin gene was used as the functional gene to correct the dystrophic phenotype in muscles of *mdx *mice caused by the absence of full-length dystrophin. However, the microdystrophin product is less effective functionally at complementing dystrophin deficiency than is the full-length cDNA, which is also a shortcoming of AAV-vector-mediated gene delivery for DMD. Nevertheless, our results suggest that VP22 mediated plasmid gene transfer is a feasible gene therapy for DMD. However, careful investigation of VP22 transport mechanisms should be conducted before its application with gene therapy since there is little know about the *in vivo *distribution and transport properties of VP22.

Although controversy remains about the trafficking potential of VP22, we demonstrate the feasibility and efficiency of VP22-mediated intercellular microdystrophin protein delivery following *in vitro *and *in vivo *gene transfection, suggesting that the VP22-mediated protein delivery system can be a useful tool to enhance the efficacy of DMD gene therapy.

## Conclusion

Overall, our results clearly demonstrate that the VP22-microdystrophin fusion protein enhanced the expression of microdystrophin in transfected 3T3 cells and transported into myoblast (C2C12) cells from 3T3 cells. Moreover, VP22 could carry microdystrophin to translocate into neighboring muscle cells in dystrophin-deficient (*mdx*) mice. These results document the efficiency of the VP22-mediated intercellular protein delivery for potential therapy of DMD and suggest that protein transduction may be a potential and versatile tool to enhance the effects of gene delivery for somatic gene therapy of DMD.

## Methods

### Vector construction

Full-length cDNA for HSV-VP22 was amplified by polymerase chain reaction from plasmid pSINrep5-VP22 (kindly provided by Dr Wu T. C., Department of pathology, Johns Hopkins Medical Institutions, Baltimore, Maryland) and subcloned into the *Bam*HI/*Not*I digested multi-cloning site of pVAX1 expression vector (Invitrogen), named pAVP22. The microdystrophin cDNA (ΔR4-R23/ΔCT) is a truncated version of the full-length dystrophin cDNA which was generated by introducing deletions encoding repeats 4 though 23 within the rod domain and the cterminal domain [30]. Full-length cDNA for human microdystrophin gene was acqured from the plasmid pBSK-MICRO digested with *Not*I to release the 3.75 kb fragment containing human microdystrophin gene, and this piece was subcloned into the *Not*I sites of pAVP22 (C-terminal fusion to VP22) and pVAX1 respectively, named pAVP22-MICDYS and pAMICDYS, pVAX1 vectors are driven by the CMV enhancer/promoter.

### Cell culture and transfection

Mouse embryo fibroblast 3T3 cell lines and mouse C2C12 myoblasts were maintained in a humidified incubator at 37°C and 5% CO_2 _in Dulbecco's modified Eagle medium (DMEM) containing 10% fetal calf serum, 4.5 g/l glucose, 3.7 g/l NaHCO3, and 1% penicillin and streptomycin (Gibco). The 3T3 cells were grown in six-well polystyrene tissue culture dishes, after growen to more than 80%~90%, cells were individually infected with 3.0 μg of expression plasmid DNA (pAVP22, pAMICDYS, pAVP22-MICDYS) in OPTI-MEM using the Lipofectamine™ 2000 Transfection Reagent Kit (Invitrogen) according to the manufacturer's specifications. Forty-eight hours after transfection, the media supernatant was collected and filtered, and then the media supernatant was transferred onto the C2C12 myotubes. After transferred for 45 min, the expression of microdystrophin was detected by immunocytochemistry.

### Immunofluorescence staining

Transfected cells were fixed with 4% paraformaldehyde/PBS, followed by 100% methanol, and then rinsed twice with PBS and blocked by 5% HS for 1 h. Next, the cells were incubated overnight at 4°C with the primary antibodies mouse anti-dystrophin (1:20, Amino Terminal, Chemicon). After thee rinses with PBS, CY_3_-conjugated goat abti-mouse IgG (1:200, sigma) was added as a secondary antibody, and the cells were incubated for 1 h at room temperature. Finally, all cells were rinsed thee times with PBS, cell nuclei were counterstained with 0.01% 4', 6-diamidino-2-phenylindole (DAPI) (Sigma) for 5 min, and then viewed with a fluorescent microscope (Olympus BX51). The microdystrophin-expressing positive cells were counted and expressed as the average number of positive cells per 6-well plate.

### Animal injection

The mdx mice used in this study were originally purchased from The Jackson Laboratory (Bar Harbor, ME, USA). The colonies were subsequently established by in-house breeding at the Laboratory Animal Center of Sun-Yet University and mice (including experimental mice and breeding pairs) were housed in a specific-pathogen-free animal facility. All animal experiments were accorded to Sun-Yet University guidelines for animal care. All mdx mice were injected at the age of 6–8 weeks with the plasmid DNA (pAVP22-MICDYS, pAMICDYS) diluted in PBS (1 μg/μl) though tow routes (i.m. and i.p.): 100 μl of plasmid DNA was injected directly into the left TA muscles, as the control,100 μl PBS was injected directly into the right TA muscles; 300 μl of plasmid DNA was injected though the intraperitoneally injection (Along the diaphragm) respectively. Before the injection, the mdx mice were anesthetized by intraperitoneal injection of 1.2% Avertin (0.24 mg/g body weight). All tissues were harvested after different time points of the injection with plasmid DNA. The mice were euthanized by an overdose of pentobarbital, all tissue samples were snap-frozen in liquid nitrogen and processed for subsequent immunocytochemical, molecular detection.

### Measurement of diaphragm muscle contractile properties

After the mice were killed by an overdose of pentobarbital, a strip of the ventral part of the costal diaphragm with the surrounding rib cage was carefully dissected out from the muscle in situ and immediately placed in the oxygenated Ringer's solution (120 mM NaCl, 5.0 mM KCl,1.0 mM CaCl_2_, 1.0 mM KH_2_PO_4_, 1.0 mM MgSO_2_, 10.0 mM glucose, and 10.0 mM HEPES, corrected to a pH of 7.40). And then the freshly diaphragm strip was rapidly mounted vertically in a tissue chamber(HW-400SE, Co.ltd.TaiMeng Technology, China) filled with a Ringer's solution perfused with 95%O_2_:5%CO_2_. and maintained at 25°C [39]. The costal end of the diaphragm strip was held in a clip at the bottom of the chamber, whereas the central tendon end was maintained with a second clip which was attached to an electromagnetic force-transducer device (BL-420E+, Co. Ltd. TaiMeng Technology, China). After a 20-min equilibration period, we adjusted the muscle strip to optimal length L0 (The muscle length that allowed maximal twitch force to be achieved was defined as L0), and then the muscle was supramaximally stimulated via two platinum electrodes arranged longitudinally on either side of the muscle, which were attached to a a square wave pulse stimulator BL-420E+ Stimulator (Co. Ltd. TaiMeng Technology, China). A force frequency curve was determined by stimulating muscle strips at 10,20,40,60,80, 100,120,140 and 160 Hz (1-ms pulse duration, 330-ms train duration, 10 per minute). The peak force generated by this protocol was used to calculate the maximum tetanic force. Stimulations were controlled, and data were collected and analyzed with the use of custom software developed in Co. Ltd. TaiMeng Technology (BL-420E+ experimental system with the function of biology). Specific twitch and tetanic force normalized to muscle CSA (kN/m^2^) were calculated according to the following equation: CSA = (muscle mass, g)/[(optimal fiber length, cm) × (muscle density, g/cm^3^)]. A muscle density estimated at 1.06 g/cm [40]. All measurements were performed on treated and untreated diaphragm strips of mdx mice as well as in untreated diaphragm strips obtained from wild-type C57/BL10 mice of the same age.

### HE staining and central nucleation quantification

TA muscles samples were snap frozen in liquid nitrogen-cooled isopentane and then made the frozen sections stored in -80°C. Doing the HE staining to the ice slice of the muscular tissue of experimental mice. The percentage of centrally nucleated myofibers in the mdx mouse TA muscle was determined by manually counting the total number of myofibers and the total number of myofibers carrying centrally located nuclei in a 6 μm HE-stained section with an electronic colony counter. Equal or more than four representative muscle sections were quantified for each muscle sample. Calculating the percentage of central nucleated fibers in whole observation fibers in 5 continuous scopes. The percentage of central nucleation was calculated with the formula % central nucleation = (total number of myofibers carrying centrally located nuclei)/(total number of myofibers).

### Indirect immunofluorescence detection of microdystrophin gene expression

Cryostat sections (6 μm) were prepared from the snap frozen muscle samples. Slides were blocked with PBS containing 10% normal goat serum for 1 h. Subsequently sections were incubated with anti-dystrophin antibody, a mouse monoclonal antibody directed against a amino-terminal polypeptide fragment of human dystrophin (1:20, Chemicon) at 4°C for over night. The secondary antibody, FITC-conjugated polyclonal antibody goat-ant-mice (1:200, Sigma) were applied for 1 h. The sections were viewed with a fluorescent microscope. The microdystrophin expressing positive fibers were counted and expressed as the average number of positive fibers per muscle cross-section. At least four sections were counted for each muscle. The positive stain fibers were observed for successive 10 visual fields with fluorescent microscope. Count the percentage of microdystrophin positive fibers in the whole observing fibers and take the pictures to these fibers. Muscle cell nuclei were counterstained with 0.01% 4', 6-diamidino-2-phenylindole (DAPI) (Sigma) for 5 min. Photographs were taken with an Olympus IX71 fluorescent microscope.

### Western blot analysis

Total cellular protein was extracted with a reducing sample buffer (10% SDS, 70 mM Tris-HCl, pH 6.8, 5% b-mercaptoethanol, and 10 mM EDTA) from the tibialis anterior (TA) muscle. The protein concentration was determined by Bio-Rad protein assay. Protein/lane was separated on a 6% SDS-polyacrylamide gel and electrically transferred to a PVDF membrane (Millipore). After blocking with 3% skim milk, the blot was incubated with a mouse anti-dystrophin (amino terminal) monoclonal antibody (1:30 dilution, Chemicon). After washing with TBST, the blot was incubated with a 1:5000 dilution of HRP-conjugated rabbit anti-mouse IgG1 antibody (Chemicon). The signal was detected by using the enhanced chemiluminescence method (Hyperfilm ECL; Amersham Pharmacia Biotech, UK).

### Statistical analyses

Values in the text and tables are reported as mean ± SEM. Variables were compared between experimental groups using Student's *t *test. Differences between groups were considered significant when *P *was < 0.05.

## Authors' contributions

Fu Xiong and Shaobo Xiao collectively conceived the study, designed the experiment. Fu Xiong and Meijuan Yu completed the functional assessment *in vivo*, prepared the figures for the manuscript and wrote the manuscript. Shaobo xiao and Liurong Fang completed the functional assessment *in vitro*. Fu Xiong and Wanyi Li carried out the Western blot for human microdystrophin and assisted to animal injection, and Meijuan Yu guided other experiments such as HE, immunochemistry *ect*. Hui Zheng, Yanchang Shang and Funing Peng carried out HE and indirect immunofluorescence testing. Cuiping Zhao and Wenliang Zhou completed the measurement of diaphragm muscle contractile properties. Jeffrey S. Chamberlain offered the plasmid pBSK-MICRO and revised the manuscript. Cheng zhang and Huanchun Chen contributed to the conception and design of the experiment and were also involved in editing the manuscript. All of the authors have read and approved the final manuscript.
